# An Amperometric Acetylcholine Biosensor Based on Co-Immobilization of Enzyme Nanoparticles onto Nanocomposite

**DOI:** 10.3390/bios13030386

**Published:** 2023-03-15

**Authors:** Jyoti Ahlawat, Minakshi Sharma, Chandra Shekhar Pundir

**Affiliations:** 1 Department of Zoology, Maharshi Dayanand University, Rohtak 124001, India; jyotiahlawat2608@gmail.com (J.A.); sminakshi.2007@rediffmail.com (M.S.); 2 Department of Biochemistry, Maharshi Dayanand University, Rohtak 124001, India

**Keywords:** acetylcholinesterase nanoparticles, choline oxidase nanoparticles, biosensor, serum, Alzheimer’s

## Abstract

An electrochemical biosensor was fabricated using nanoparticles of acetylcholinesterase (AChE) and choline oxidase (ChO)/Pt nanoparticles (PtNPs)/porous graphene oxide nanosheet (GONS) composite. A pencil graphite electrode (PGE) was used for the electrodeposition of nanocomposite and the determination of acetylcholine (ACh), a neurotransmitter. Various techniques such as scanning electron microscopy (SEM), transmission electron microscopy (TEM), X-ray diffraction (XRD), Fourier-transform infrared (FTIR) spectra and cyclic voltammetry (CV) were used for characterization. This biosensor (AChENPs-ChONPs/GONS/PtNPs/PGE) indicated a very short response time (3 s), a lower limit of detection (0.001 µM), good linearity (0.001–200 µM), longer storage stability (6 months) and better reproducibility. The percent analytical recoveries of added acetylcholine in serum (5.0 and 10 µM) were found to be 97.6 ± 0.7 and 96.5 ± 0.3 for the present biosensor. The coefficients of variation were obtained to be 8% and 3.25%, correspondingly. The biosensor was applied to measure the ACh amount in the serum of healthy individuals and patients with Alzheimer’s disease. The number of interferents had no effect on the biosensor at their physiological concentrations.

## 1. Introduction

Neurotransmitters are substances that the body utilizes to communicate between cells. In the case of the human body, acetylcholine (ACh) is one of the most common neurotransmitters. It is secreted by cholinergic neurons. Choline (Ch) and its coenzyme A ester (acetyl CoA) are converted to ACh (CH_3_COOCH_2_CH_2_N^+^ (CH_3_)_3_ by an enzyme known as acetylcholine transferase. ACh, scientifically referred to as 2-acetoxy-N, N, N-trimethylethanaminium, is an important neurotransmitter in which both the central and peripheral nervous systems (PNS) are involved [1]. Henry Hallett Dale first discovered it in 1914, and Otto Loewi subsequently verified its existence in 1922. Nearly one-hundredth of the world’s population is affected by neurological illnesses, according to the World Health Organization (WHO) [2]. Medications that interfere with ACh, a neurotransmitter critical to muscular activity, may induce varying degrees of movement disturbances or even paralysis. Functions that are impacted by ACh are cognition, memory, sleep, focus, and learning capacity.

ACh levels in neurological disorders can be determined by using a variety of procedures, together with an improved Elman colorimetric test for cholinesterase activity estimation [3], capillary electrophoresis, capillary zone electrophoresis, ion-sensitive field effect transistors (ISFETs), high-performance liquid chromatography (HPLC) [4,5], mass spectrometry [6], hydrophilic interaction chromatography [7], etc. However, the majority of these methods are complex, costly and time-consuming, with less sensitivity and selectivity. For the clinical diagnosis of acetylcholine, biosensing methods are more efficient, accurate, time-saving, specific, rapid and portable.

Electrochemical biosensors based on the co-immobilization of enzymes onto different electrode surfaces have been reported [8,9,10,11,12,13,14,15,16,17,18,19]. These electrochemical biosensors convert biological signals into measurable signals [20,21]. In these earlier biosensors, direct immobilization of AChE-ChO enzymes on the working electrode could cause a loss of the activity of the native enzyme [22]. Therefore, to overcome this problem, enzyme nanoparticle (ENP)-based biosensors have been used, which have shown better analytical performance and response [23,24,25].

PG electrodes are better than other electrodes such as Au E, GCE, Pt E, CPE, FTO, SPCE, ITO, etc., due to their improved stability, reproducibility, low cost, durability and low background noise, as well as being more economical and stiffer [26,27].

Likewise, other noble metal nanoparticles (gold and silver) have similar characteristics to platinum nanoparticles (PtNPs) in terms of more surface area, biocompatibility, better conductivity, good electrocatalytic features for oxidation/reduction toward H_2_O_2_, etc. [28]. In combination with other nanoparticles, these metal nanoparticles show enhanced properties such as increased surface area, capacity to endorse the transfer of electrons and virtuous catalytic activity for H_2_O_2._

Graphene is a 2D carbon material having different arrangements such as graphene oxide (GO) and reduced graphene oxide/graphene nanosheets. Characteristics such as large surface area, thermal stability, mechanical properties, conducting properties, electrochemical behavior, etc., were shown [29]. Therefore, graphene oxide nanosheets are increasingly considered for the immobilization of enzymes and for biosensor use [30].

Features such as better conductivity, selectivity and sensitivity have been shown by the fabrication of nanomaterials onto PG electrodes [31]. During the planning and optimization of biosensors, great interest has been shown in the fabrication of enzyme nanoparticles onto modified electrodes. An electrochemical biosensor using enzyme nanoparticles for the detection of acetylcholine has not been reported so far. Compared to other methods, these biosensing methods are simple, sensitive and specific. Enzyme-nanoparticle-based biosensors are more sensitive and specific. Therefore, in the present work, we used nanoparticles of AChENPs/ChONPs onto graphene oxide nanosheet (GONS)/PtNPs nanocomposite-modified PG as a working electrode for the determination of acetylcholine in different samples of blood serum.

## 2. Materials and Methods

### 2.1. Materials

Acetylcholine esterase (EC 3.1.1.7) was obtained from electric eel. Choline oxidase (EC 1.1.3.17) was obtained from Alcaligenes sp. Acetylcholine chloride, hexachloroplatinic acid, graphite powder, acetone, sodium nitrate, concentrated sulfuric acid, potassium permanganate, ascorbic acid, ethanol and methanol were obtained from Sigma Aldrich (St. Louis, MO, USA). A 6B pencil (Make: Apsara) with graphite lead of 2 mm thickness was obtained from a local shop and used as the working electrode. In all experiments, deionized water (DW) and analytical reagent (AR)-grade chemicals were used. Serum samples were collected from the hospital of PGIMS, Rohtak.

### 2.2. Apparatus

A potentiostat instrument designed by AutoLab (Model: AUT83785; Manufacturer: Ecochemie, The Netherlands) was used. It comprised three electrodes, i.e., Pt wire as the auxiliary electrode, a Ag/AgCl electrode (3.5 M KCl) as the reference electrode and AChENPs/ChONPs/PtNPs/GONS/PGE as the working electrode. Fourier-transform infrared spectroscopy (FTIR) (manufactured by Bruker), a JEM 2100-F Transmission electron microscope (TEM) (manufactured by JEOL, Japan) and a D/Max 2550 X-ray diffractometer (XRD) (manufactured by Rigaku, Tokyo, Japan) were used.

### 2.3. Synthesis of Platinum Nanoparticles (PtNPs)

The preparation of PtNPs was performed as described by Thirumurugan et al. [32], with minor modifications. To synthesize PtNPs, 10 mL of leaf extract of citrus lemon was added to 190 mL of 1 mM aqueous chloroplatinic acid extract (H_2_PtCl_6_.6H_2_0) under continuous stirring. After complete addition of the leaf extract, the mixture was kept for incubation at 30 °C for 24 h. The color of the solution changed from light yellow to dark brown, which revealed the synthesis of PtNPs. The colored solution was centrifuged for 30 min, followed by redispersion in DW to remove further unwanted biological impurities.

### 2.4. Preparation of Porous Graphene Oxide Nanosheets (GONS)

Firstly, the improved Hummer method [33] was used for the preparation of graphene oxide (GO) as follows: 0.5 g of pencil graphite powder, 0.50 g of sodium nitrate and 25 mL of sulfuric acid (H_2_SO_4_ 98%) were mixed in a reaction flask of 500 mL under continuous stirring for 15 min in a water bath with ice at a temperature between 0 and 5 °C. Then, 4.0 g of potassium permanganate was added slowly to the above solution within 15 min at 20 °C. Following that, DW (20 mL) was added slowly under stirring at 40 °C for 90 min in the water bath. A brown-colored suspension was formed, which was treated with 30% H_2_O_2_ solution (6 mL). A 5% amount of hydrochloric acid (HCl) was used to wash the GO suspension and was monitored by DW for the removal of excess manganese (Mn) until it reached neutrality. An oven was used to dry the purified GO at 60 °C for 24 h. To prepare the graphene oxide nanosheet (GONS), GO (0.1 g) and broccoli juice (240 µL) were added to a beaker (250 mL), producing a homogeneous dispersion. This dispersion was stirred for 24 h. After stirring, it was sonicated for 60 min and, lastly, dried at 60 °C for 24 h.

### 2.5. Preparation of AChENPs/ChONPs

For the preparation of AChENPs/ChONPs, the desolvation method was used by using ethanol [34]. Firstly, the enzyme (acetylcholinesterase/choline oxidase separately) was dissolved in DW (2 mg/mL). Then, 4 mL of absolute ethanol was added dropwise (0.1 mL/min) under continuous stirring (500 rpm). To confirm the complete cross-linking of the respective enzyme nanoparticles (ENPs), 1.8 mL of glutaraldehyde solution (2.5%) was added to the solution under the same stirring settings at 4 °C for 24 h. The differential centrifugation technique was used for the precipitation of ENPs from two suspensions at 12,000× *g* (min, 4 °C). After that, a 0.12 g amount of cysteamine di-hydrochloride was added to the ENPs under continuous stirring (5–6 h), which provides an amino group to the ENPs. These functionalized ENPs were separated from the enzyme solution by centrifugation at 1200 rpm for 10 min and stored at 4 °C until use. The size and shape of the functionalized AChENPs/ChONPs were studied by TEM from AIRF commercially.

### 2.6. Preparation of GNs/PtNPs/PG Electrode

**Figure 1:**
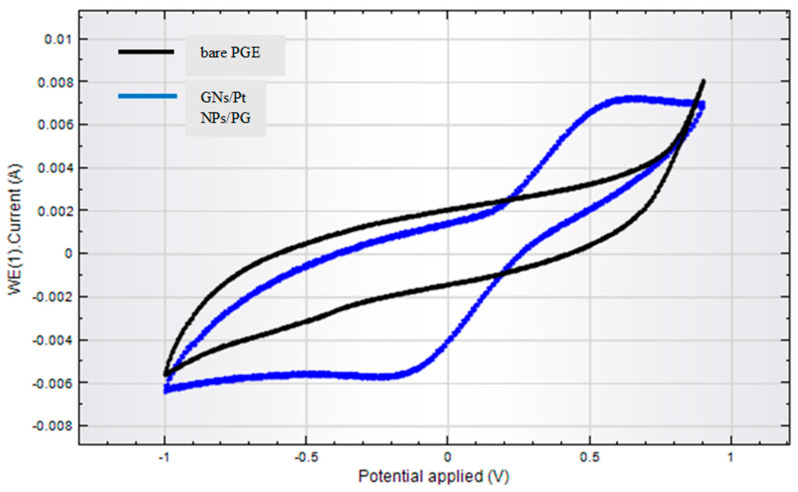
Cyclic voltammogram (CV) curve in 2.5 mM K_3_Fe(CN)_6_/K_4_Fe(CN)_6_ (1:1) with 40 continuous polymerization circles (−1.1 to 0.1 V) at a scan rate of 20 mV/s for bare PGE and GONS/PtNPs/PGE. (GONS = Graphene oxide nanosheets; PtNPs = Platinum nanoparticles; PGE = Pencil graphite electrode).

Firstly, the surface of the PG electrode (2 cm × 5 mm) was manually polished with alumina slurry by using a polishing cloth. It was monitored by systematic washing with DW. The cleaned electrode was dipped in 4 mL of piranha solution (3H_2_SO_4_:1H_2_O_2_) for 10 min and then washed with DW. A PtNPs/GONS hybrid was prepared by taking both in an equal ratio (2 mg/2 mL) under continuous stirring for 12 h, followed by centrifugation at 1200 rpm to separate the nanocomposite (PtNPs/GONS). Cyclic voltammetry in a potentiostat/galvanostat was used for the electrodeposition of PtNPs/GONS on the surface of the PGE by immersing it in 23 mL of 2.5 mM K_3_Fe(CN)_6_/K_4_Fe(CN)_6_ (1:1) and 2 mL of PtNPs/GONS solution with 40 continuous polymerization circles (−1.1 to 0.1 V) at a scan rate of 20 mV/s. The subsequent PtNPs/GONS-modified PGE was washed thoroughly with DW to get rid of boundless matter (Figure 1).

### 2.7. Co-Immobilization of AChENPs/ChONPs onto PtNPs/GONS-Modified PG Electrode

**Figure 2:**
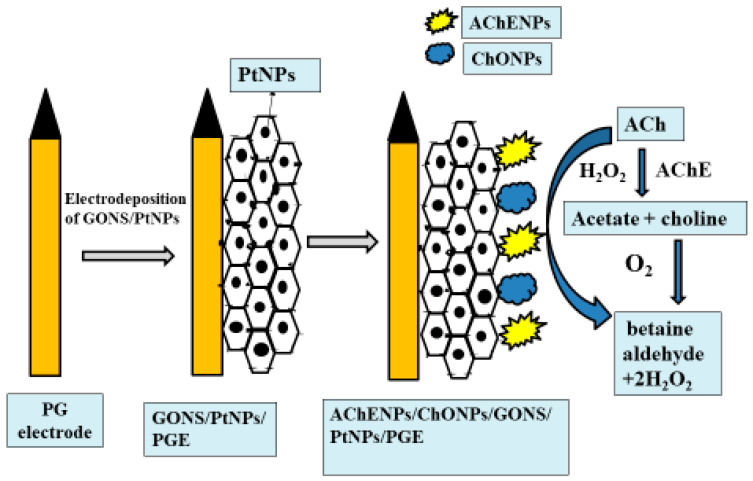
Schematic representation showing the construction of acetylcholine biosensor based on AChENPs/ChONPs/GONS/PtNPs/PGE. (AChENPs = Acetylcholine esterase nanoparticles; ChONPs = Choline oxide nanoparticles; GONS = Graphene oxide nanosheets; PtNPs = Platinum nanoparticles; PGE = Pencil graphite electrode).

The GONS/PtNPs/PGE was immersed into 4 mL of AChENPs + ChONPs suspension for 24 h at 4 °C. The resulting AChENPs/ChONPs/GONS/PtNPs/PG electrode was washed 3–4 times with DW to remove boundless AChENPs and ChONPs. The AChENPs/ChONPs/GONS/PtNPs/PGE was used as a working electrode, as shown in Figure 2, and stored at 4 °C while not in practice. The working electrode was characterized by means of SEM and FTIR before and after immobilization of AChENPs and ChONPs.

### 2.8. Construction of Amperometric Acetylcholine Biosensor

An amperometric acetylcholine biosensor was constructed using AChENPs/ChONPs/GONS/PtNPs/PGE (working electrode), Pt wire (counter electrode) and Ag/AgCl (reference electrode) saturated 3.5 M KCl. All measurements were performed on an electrochemical analyzer (AutoLab PGSTAT 30) at room temperature. The cyclic voltammetry experiments were performed in 25 mL of 2.5 mMK_3_Fe(CN)_6_/K_4_Fe(CN)_6_ (1:1) with 200 µL of AChCl (0.05 M), and a voltage range between −0.1 and +0.1 V at a scan rate of 20 mV/s was applied. Hence, the working of this biosensor is based on the following reaction:



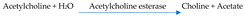





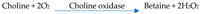





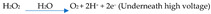



The current measurement or flow of electrons in the galvanostat remains directly related to the acetylcholine concentration.

### 2.9. Optimization of Acetylcholine Biosensor

The effects of various analytical parameters such as pH, incubation temperature, substrate (acetylcholine) concentration and reaction period were considered for the optimization of the AChENPs/ChONPs/GONS/PtNP-modified PGE-based nanosensor. For the determination of optimum pH, phosphate buffers (0.1 M) with pH between 5.0 and 10 at a break of 0.5 were prepared. Similarly, the incubation temperature range (20–50 °C) at an interval of 5 °C in a temperature-controlled water bath and at a time duration of 2–90 s was measured for the detection of the most favorable temperature and incubation time, respectively. The acetylcholine effect on the biosensor response was studied by changing the concentration of acetylcholine in the range of 0.001–200 µM.

### 2.10. Evaluation of Acetylcholine Biosensor

The acetylcholine biosensor was evaluated by measuring its lower detection limit (LOD), % analytical recovery of added acetylcholine in serum, within and between batch coefficients of variation (precision) and correlation with the standard method.

## 3. Results and Discussion

### 3.1. Transmission Electron Microscope (TEM) Study

**Figure 3:**
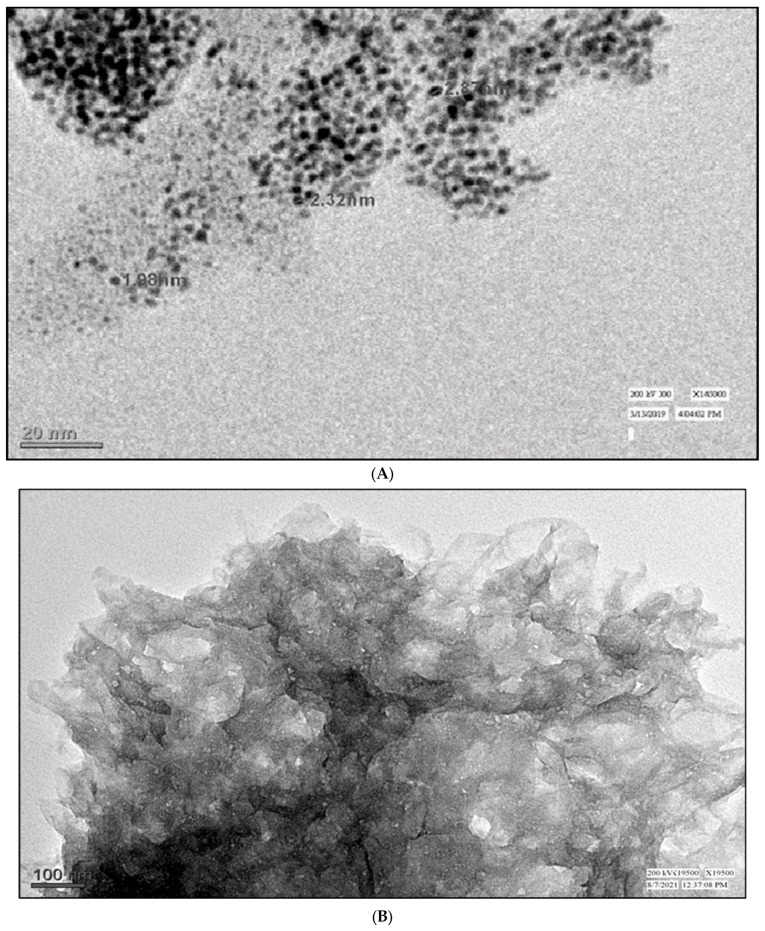

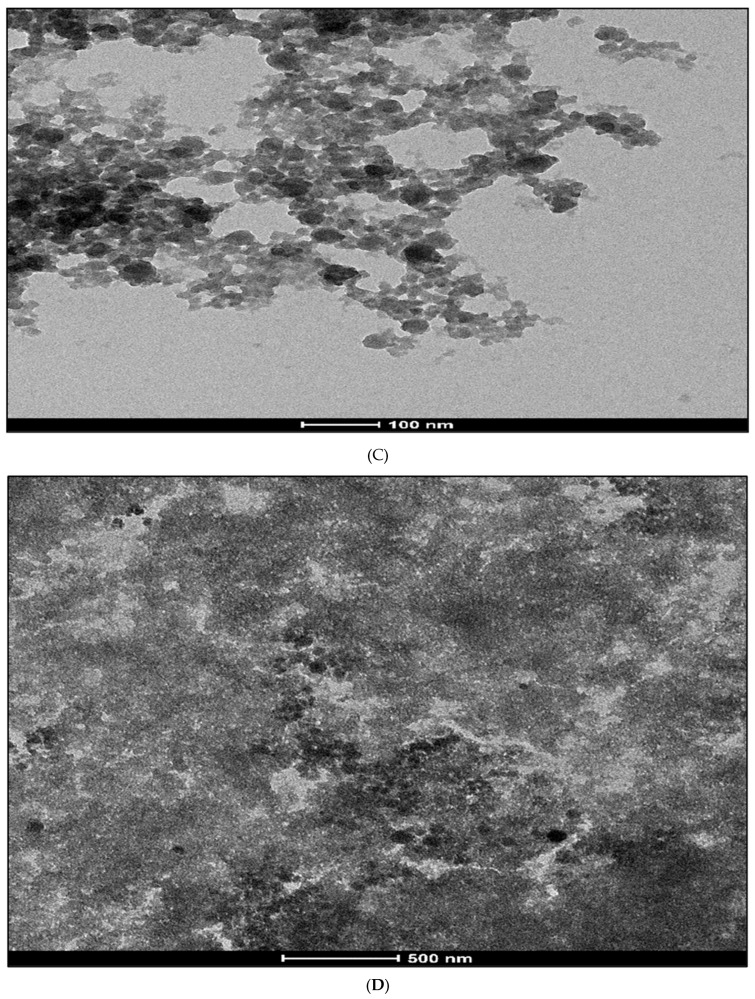
Transmission electron microscopy (TEM) images of (**A**) PtNPs, (**B**) GONS, (**C**) AChENPs and (**D**) ChONPs. (AChENPs = Acetylcholine esterase nanoparticles; ChONPs = Choline oxide nanoparticles; GONS = Graphene oxide nanosheets; PtNPs = Platinum nanoparticles).

The TEM study revealed that the mean size of the PtNPs is 20 nm, and they are predominantly spherical in shape, while some of the nanoparticles have a dodecahedron shape, as shown in Figure 3A. The TEM image shown in Figure 3A confirms thin, porous graphene sheets with a wrinkled-like material and crumpled ends (Figure 3B). The average size of AChENPs and ChONPs was in the range of 1–100 nm in diameter, as studied by taking their images in TEM, as shown in Figure 3C,D. The diameter of further ENPs was also described similarly [35].

### 3.2. X-ray Diffraction (XRD) Study

**Figure 4:**
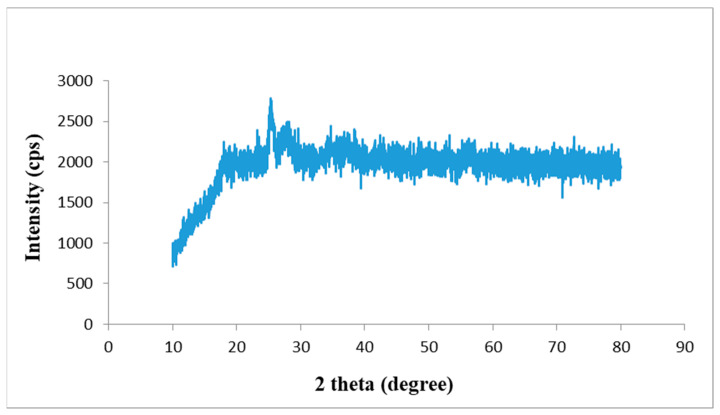
X-ray diffraction (XRD) of GONS. (GONS = Graphene oxide nanosheets).

The XRD study revealed a sharp diffraction peak at 2θ = 25°, which indicated that the porous graphene nanosheets existed individually with a highly disordered nature (Figure 4).

### 3.3. Fourier-Transform Infrared (FTIR) Study

**Figure 5:**
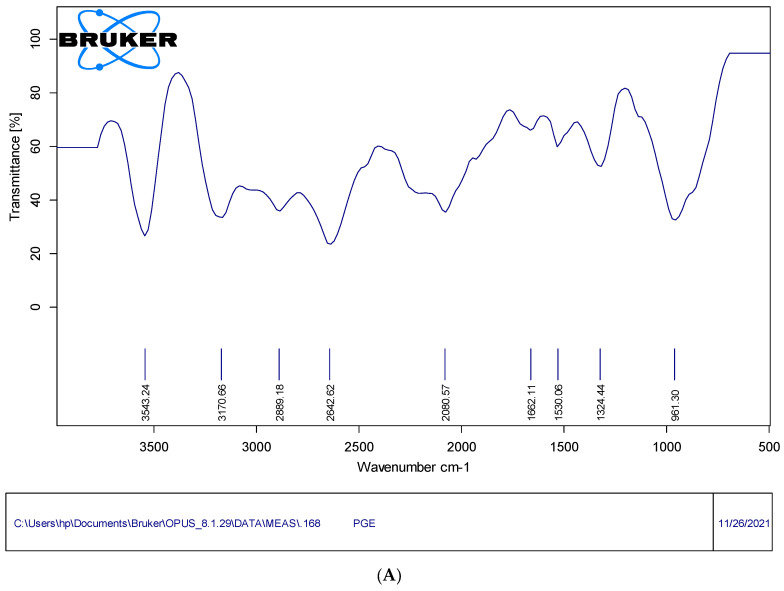

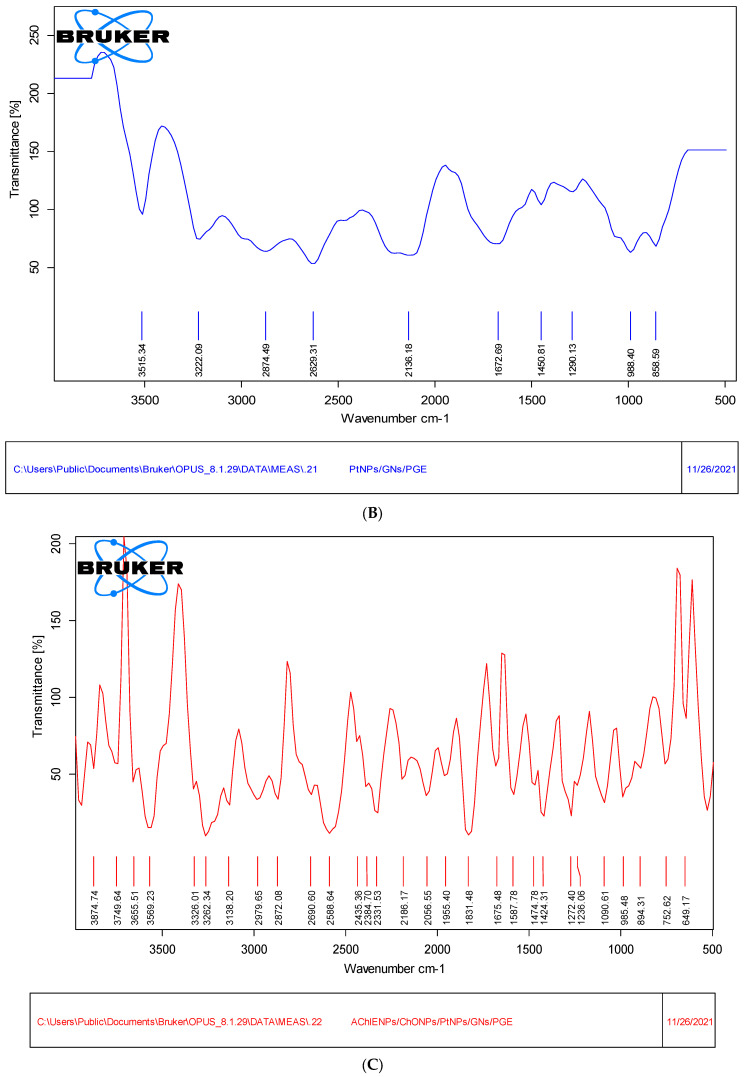
Fourier-transform infrared spectra of (**A**) bare PGE, (**B**) GONS/PtNPs/PGE and (**C**) AChENPs/ChONPs/GONS/PtNPs/PGE in the range 4000–500 cm^−1^. (AChENPs = Acetylcholine esterase nanoparticles; ChONPs = Choline oxide nanoparticles; GONS = Graphene oxide nanosheets; PtNPs = Platinum nanoparticles; PGE = Pencil graphite electrode).

The FTIR analysis of PtNPs showed a broad band at 3341 cm^−1^, which was owed to the N-H enlarging vibration of the -NH_2_ group. The value at the 2332 cm^−1^ wavelength corresponds to C-N stretching, and the strong intense peak value at 1634 cm^−1^ may be due to the presence of –C=C stretching. The peaks at 1173 cm^−1^ were due to C-O bonding, as shown in Figure 5A. The spectrum of porous graphene nanosheets is represented by the peaks at 809 cm^−1^, 1636 cm^−1^, 2344 cm^−1^ and 3309 cm^−1^, resulting from C-H bonding, C=C conjugation, C-N band and N-H extending vibrations. The lack of bands corresponding to the oxygen-functional groups in the porous graphene oxide nanosheet spectrum further confirms its formation (Figure 5B). The bare PG electrode showed peaks at 1530 cm^−1^ and 1632 (C=C bonding), 2080 cm^−1^ and 2462 cm^−1^ (C≡C extending), and 3170 cm^−1^ (C-H bonding), as shown in Figure 5C. The PtNPs/GNs/PG electrode showed bands at 988 cm^−1^, 1290 cm^−1^, 1632 cm^−1^ and 3222 cm^−1^, which confirms O-H bonding, C-N stretching and N-H, as well as C-H bonding. The AChENPs/ChONPs/PtNPs/GONS/PG electrode showed various bonds at different peaks of 1236–1955 cm^−1^, 2056–2979 cm^−1^ and 3138–3874 cm^−1^, which confirms the presence of enzyme nanoparticles.

### 3.4. Scanning Electron Microscope (SEM) Study

**Figure 6:**
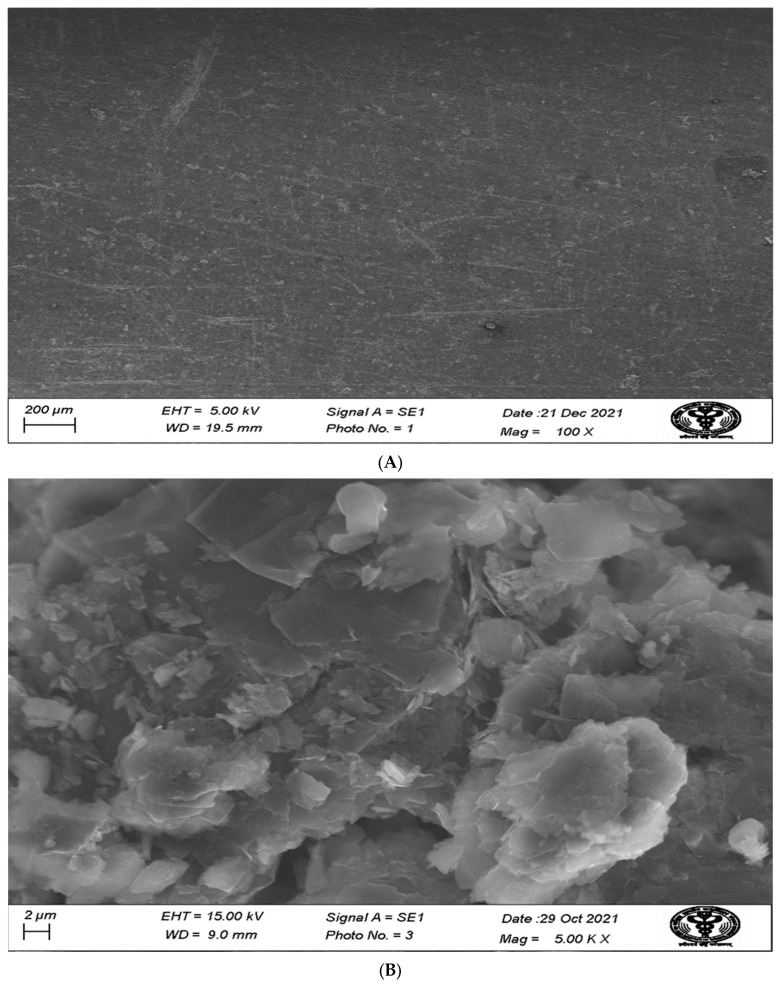
Scanning electron microscopy (SEM) image of bare PG electrode (**A**) and AChENPs/ChONPs/GONS/PtNPs nanocomposite onto PG electrode (**B**). (AChENPs = Acetylcholine esterase nanoparticles; ChONPs = Choline oxide nanoparticles; GONS = Graphene nanosheets; PtNPs = Platinum nanoparticles; PGE= Pencil graphite electrode).

The bare electrode studied by SEM shows no deposition (Figure 6A) onto PGE, whereas the AChENPs/ChONPs/GONS/PtNP-modified PGE depicts the deposition of nanoparticles, as it confirmed the co-immobilization of AChENPs and ChONPs (Figure 6B). Some earlier studies reported the co-immobilization of ENPs onto the PGE [33].

### 3.5. Optimization of Acetylcholine Biosensor

**Figure 7:**
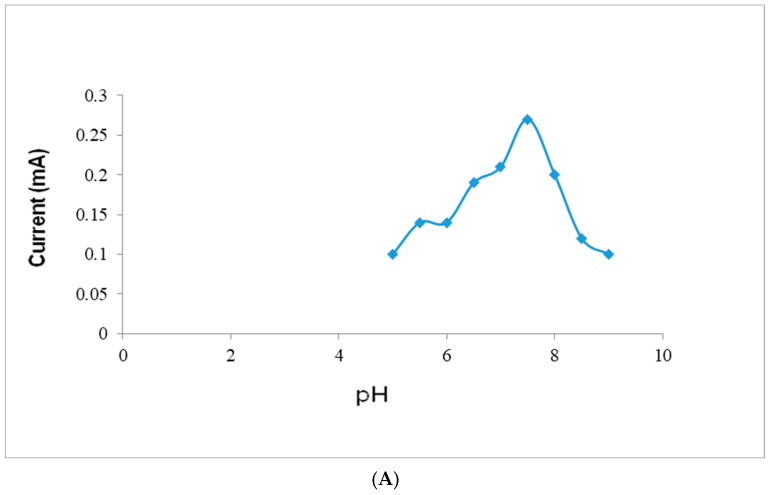

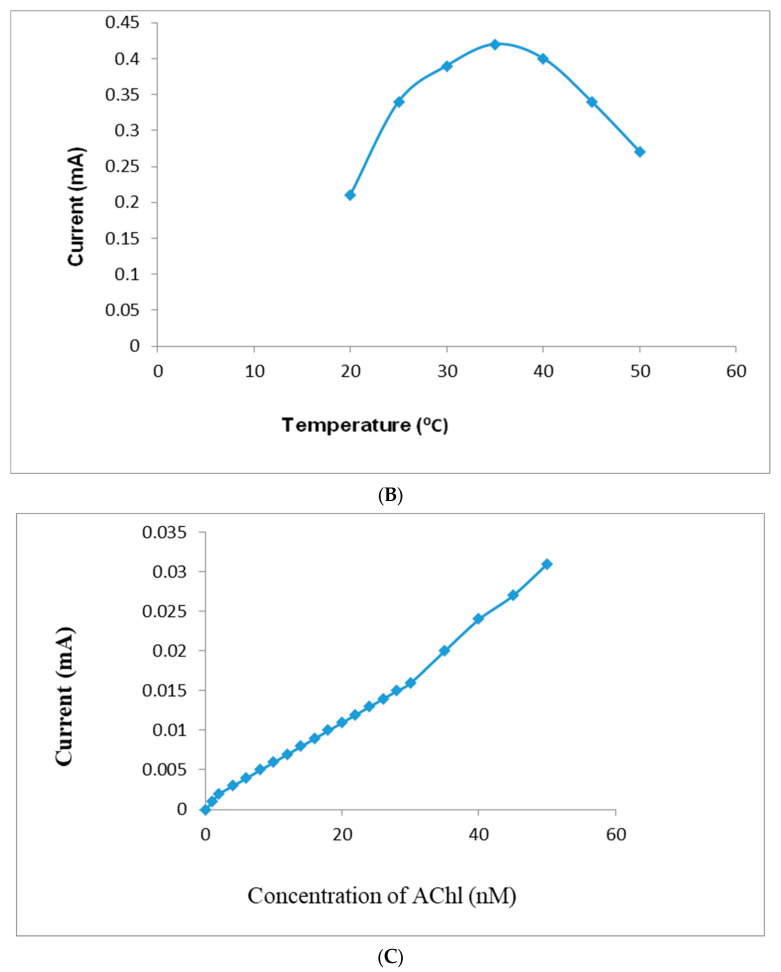
(**A**) Influence of applied pH on the current response of AChENPs/ChONPs/GONS/PtNPs/PGE. Standard conditions of assay were used, except for the pH, which was varied as given above. (**B**) Influence of applied incubation temperature on the current response of AChENPs/ChONPs/GONS/PtNPs/PGE. Standard conditions of assay were used, except for the incubation temperature, which was varied. (**C**) Standard curve of acetylcholine concentration by acetylcholine biosensor based on AChENPs/ChONPs/GONS/PtNPs/PGE. Standard conditions of assay were used, except for the acetylcholine biosensor, which was varied as given above. (AChENPs = Acetylcholine esterase nanoparticles; ChONPs = Choline oxide nanoparticles; GONS = Graphene oxide nanosheets; PtNPs = Platinum nanoparticles; PGE = Pencil graphite electrode).

The present acetylcholine biosensor showed an optimum current at pH 7.5 (Figure 7A) and an incubation temperature of 35 °C (Figure 7B). All these optimization studies were carried out in 0.1 M sodium PB in the pH range of 5.0–10. The current response decreases at various pH and temperatures due to the slight shifting of redox peaks. Therefore, acetylcholine determination was performed in 0.1 M sodium phosphate buffer (pH 7.5; temperature, 35 °C; and maximum response potential, +0.1 V). The biosensor showed an optimum response within 4 s, which is similar to biosensors based on AChE-ChO/GrNP/PtNPs/ITO-coated glass plates [16]. The optimum pH of the present biosensor was similar to that of earlier research based on AChE-ChO/AuNPs-GO/indium tin oxide (ITO)-coated glass plates [15]; closer to the optimum pH 7.4 of AChE-ChO/metallic organic framework (MOF) and AChE-ChO/PtNPs/AuE-based biosensors [14]; higher than biosensors based on AChE-ChO/Fe_2_O_3_NPs/rGO/PEDOT/fluorine-doped tin oxide (FTO) electrodes [17], AChE-ChO/MWCNT-manganese oxide MnO_2_/rGO/gold electrodes (AuEs) [13] and AChE-ChO/CDs-APTES/CPEs [19]; but lower than AChE-ChO/quantum dots (QDs)/reduced graphene oxide (rGO)/GCE biosensors [10] and biofunctional AMs-AChE-ChO/graphene–gold nanoparticles–chitosan (GR-AuNPs-CS)/GCE [9] and AChE-ChO/polypyrrole–polyvinyl sulfonate (PPy-PVS)/platinum (Pt) electrodes [12]. The optimum temperature of the present nanosensor (35 °C) is similar to biosensors based on AChE-ChO/GrNPs/PtNPs/ITO-coated glass plates [16]. The performance of the present biosensor increased with an increase in incubation temperature, obtaining the most favorable value of 35 °C, and then further declined with a decline in temperature. The present AChENPs/ChONPs/GNs/PtNP-modified pencil graphite electrode indicated a linear increase in the current response with the rise of the acetylcholine concentration (Figure 7C).

### 3.6. Study of Scan Rate

**Figure 8:**
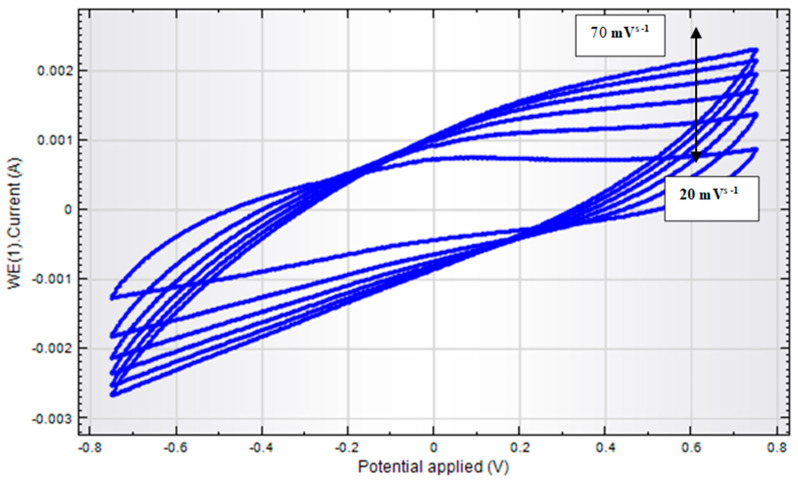
Cyclic voltammetry study of AChENPs/ChONPs/GONS/PtNPs/PGE at different scan rates for 0.1 M acetylcholine from 20 mVs^−1^, 30 mVs^−1^, 40 mVs^−1^, 50 mV^s−1^, 60 mVs^−1^ and 70 mVs^−1^. Eap = −0.8 to +0.8 V. (AChENPs = Acetylcholine esterase nanoparticles; ChONPs = Choline oxide nanoparticles; GONS = Graphene nanosheets; PtNPs = Platinum nanoparticles; PGE = Pencil graphite electrode).

The biosensor (AChENPs/ChONPs/GONS/PtNPs/PGE) showed that with varying scan rates (20, 40, 60, 80, and 100 mV s^–1^), the current response also increased accordingly, which also confirms the stability in the performance of the present biosensor (Figure 8).

### 3.7. Evaluation of Acetylcholine Nanosensor

The present AChENPs/ChONPs/GONS/PtNPs/PG electrode-based nanosensor was evaluated with different analytical parameters, such as linear range, limit of detection, coefficients of variation and analytical recovery. The present biosensor attained linearity between the current and acetylcholine (substrate) concentration range of 0.001–200 µM. By comparison with earlier biosensors, this nanosensor showed better linearity than those based on AChE-ChO/Fe_2_O_3_/rGO/PEDOT/FTO (4.0 nM–800 µM) [17], AChE-ChO/MOF/PtNPs/Au (0.01–500 µM) [14], 0.1–1.00 µM [13], and AChE-ChO/GrNP/PtNP/ITO (0.01–1000 µM) [16]. The limit of detection of the AChENPs/ChONPs/PtNPs/GNs/PG electrode was 0.1 nM, which was lower than that of earlier biosensors (0.005 µM [16], 0.1 µM, 4.0 nM [17]).

### 3.8. Analytical Recovery

The percent analytical recoveries of added acetylcholine in serum (5.0 and 10 µM) were found to be 97.6 ± 0.7 and 96.5 ± 0.3 for the present biosensor, showing the accuracy of this biosensor.

### 3.9. Reproducibility

The coefficients of variation (within and between batches) obtained were 2.8% and 3.25%, correspondingly, indicating the reliability of the present biosensor. These results also revealed the improved analytic performance of the present biosensor in terms of better repeatability and reproducibility, which can be attributed to the excellent immobilization of AChENPs/ChONPs onto the GONS/PtNPs/PG electrode.

### 3.10. Application of Acetylcholine Biosensor

The present biosensor measured the level of the acetylcholine (ACh) concentration in the serum of healthy individuals (*n* = 20), ranging from 9.0 to 12.0 nM within the normal range (8.2–11.3 nM) [17]. The level of acetylcholine in Alzheimer’s patients (*n* = 20) ranged from 1.0 to 6.5 nM, which is significantly lower (*p* < 0.01) than that in healthy individuals (Table 1). Earlier reports also revealed decreased levels of serum ACh in Alzheimer’s patients [36]

### 3.11. Correlation of Acetylcholine Biosensor

**Figure 9:**
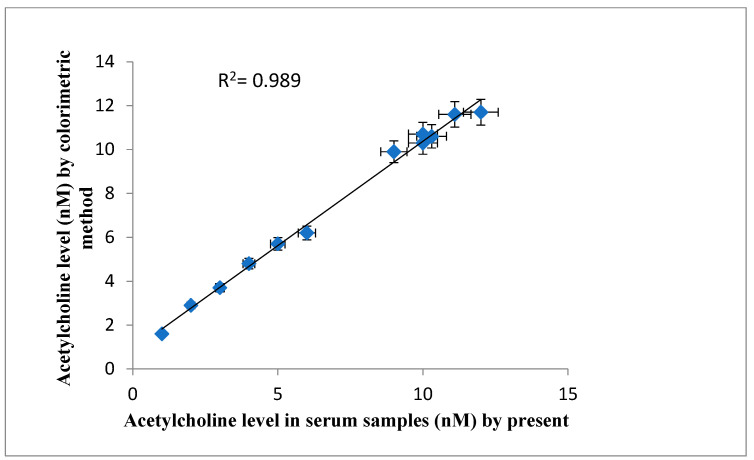
Correlation between serum acetylcholine level measured by standard method (*y*-axis) and the present method (*x*-axis) using the present acetylcholine biosensor based on AChENPs/ChONPs/GONS/PtNPs/PGE. (AChENPs = Acetylcholine esterase nanoparticles; ChONPs = Choline oxide nanoparticles; GONS = Graphene nanosheets; PtNPs = Platinum nanoparticles; PGE= Pencil graphite electrode). The line represents the correlation of acetylcholine level measused in same serum sample by the standard colorimetric method (*y*-axis) and present biosensing method (*x*-axis). The points shows the acetylcholine level in the same serum sample by the standard method and the present method with standard deviation.

The levels of ACh in serum as determined by the present biosensor were compared with those by the standard colorimetric method. The calculated correlation coefficient (R^2^ = 0.989) showed a good correlation between the existing sensor with earlier methods, as shown in Figure 9.

### 3.12. Interferents

**Figure 10:**
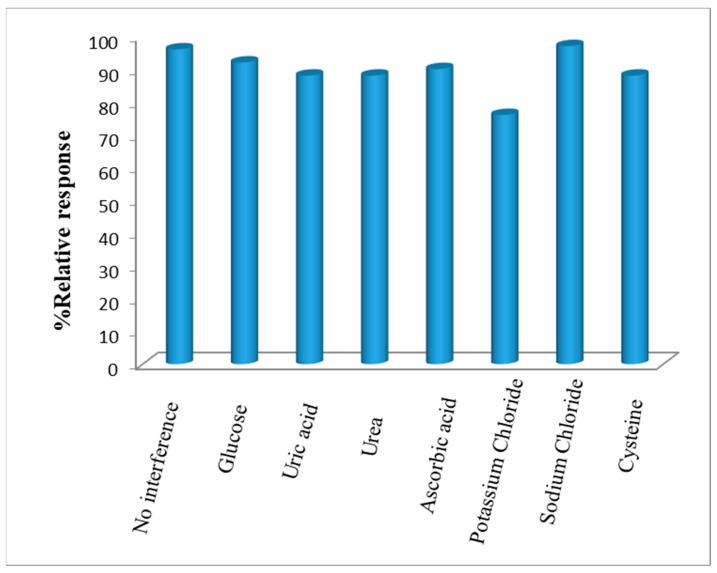
Effect of interferents (each at 0.1 mM) on the activity of AChENPs/ChONPs/GONS/PtNPs/PGE. (AChENPs = Acetylcholine esterase nanoparticles; ChONPs = Choline oxide nanoparticles; GONS = Graphene nanosheets; PtNPs = Platinum nanoparticles; PGE = Pencil graphite electrode).

The interferents study was performed amperometrically using glucose, ascorbic acid, uric acid, urea, sodium chloride, potassium chloride and L-cysteine at their physiological concentrations in 25 mL of PBS (pH 7.5). In all assays, the concentration of ACh (0.1 mM) was kept constant. The value of the current did not particularly change in the presence of these interferents. Therefore, it was concluded that the interferants impart minimal impact on the analysis of ACh by the present biosensor (Figure 10).

### 3.13. Storage Stability and Reusability of AChENPs/ChONPs/GONS/PtNPs/PGE

**Figure 11:**
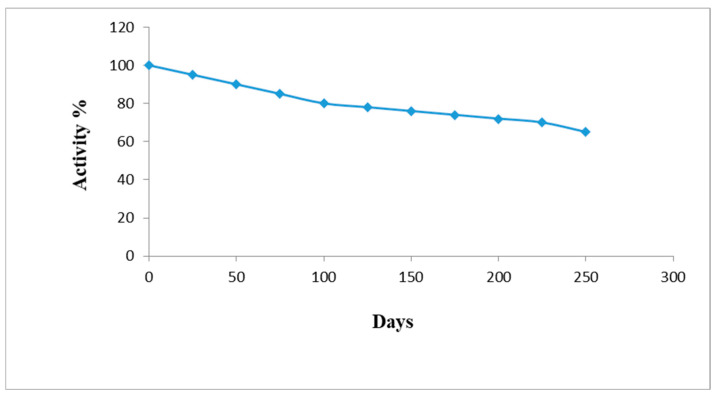
Storage stability of AChENPs/ChONPs/GONS/PtNPs/PGE at 4 °C in dry condition. (AChENPs = Acetylcholine esterase nanoparticles; ChONPs = Choline oxide nanoparticles; GONS = Graphene nanosheets; PtNPs = Platinum nanoparticles; PGE = Pencil graphite electrode).

The present nanosensor investigated the response of the acetylcholine concentration in 0.1 M PBS for a time period of 6 months. During this time, the original performance of the nanosensor decreased by 35% upon continuous use 250 times (Figure 11). Therefore, the storage stability of the present biosensor was higher than earlier biosensors based on AChE-ChO/GrNP/PtNPs/ITO (4 months) [15] AChE-ChO/MWCNT-MnO_2_/rGO/Au (3 months) [13] and AChE-ChO/ePAMAM-Sal/CPE (1 month) [19]. Various analytical parameters of the present ACh biosensor were compared with those of earlier biosensors and are summarized in Table 2.

## 4. Conclusions

An enhanced biosensor was constructed for the determination of acetylcholine in the serum samples of healthy patients and Alzheimer’s patients. The present ENP-based biosensor demonstrated better analytical performance, such as a lower detection limit (0.001 µM), shorter reaction time (3 s), good linearity (0.001–200 µM), longer storage stability (6 months) and better reproducibility, compared to earlier reports. Before this, an ENP-based biosensor for acetylcholine detection had not been prepared. Hence, this nanocomposite (GONS/PtNPs/PGE) also improved the performance of the present biosensor compared to the direct immobilization of native enzymes on the electrode surface. Therefore, it can also be used for the improvement of other biosensors.

## Figures and Tables

**Table 1:** Acetylcholine levels in serum of Alzheimer’s patients and apparently healthy individuals, as determined by biosensor based on AChENPs/ChONPs/GONS/PtNPs/PGE.

S.No.	Sex	Age (Year)	Apparently Healthy Persons (nM)	Sex	Age (Year)	Alzheimer’s Patients (nM)
1	M	50	10.0 ± 0.7	M	61	4.0 ± 0.8
2	M	56	10.5 ± 0.3	M	65	4.4 ± 0.5
3	M	48	10.7 ± 0.2	M	82	3.0 ± 0.4
4	M	52	10.4 ± 0.4	F	63	3.5 ± 0.7
5	F	60	9.2 ± 0.5	M	60	4.8 ± 0.6
6	M	40	11.1 ± 0.2	M	47	5.4 ± 0.8
7	F	65	9.0 ± 0.8	M	38	5.7 ± 0.9
8	M	43	9.7 ± 0.1	M	77	1.6 ± 0.5
9	M	48	9.6 ± 0.5	M	81	3.4 ± 0.2
10	F	68	9.3 ± 0.9	M	85	3.1 ± 0.3
11	M	38	11.1 ± 0.5	F	61	3.7 ± 0.5
12	M	36	11.7 ± 0.8	M	79	3.2 ± 0.3
13	M	25	12.2 ± 0.4	F	71	1.0 ± 0.5
14	M	44	9.9 ± 0.6	M	70	3.6 ± 0.4
15	M	39	9.2 ± 0.3	M	66	4.8 ± 0.7
16	M	27	12.0 ± 0.4	M	37	6.2 ± 0.9
17	M	30	11.7 ± 0.8	M	80	3.2 ± 0.5
18	M	37	10.5 ± 0.5	F	62	2.9 ± 0.8
19	M	29	12.1 ± 0.7	M	61	4.2 ± 0.7
20	F	55	10.3 ± 0.9	M	54	6.5 ± 0.6

**Table 2:** Comparison of various electrochemical biosensors.

Sr. No.	Composition of Electrode	Detection Method	Method of Immobilization	LOD (µM)	Linear Range (µM)	Optimum pH	Optimum Temperature (°C)	Response Time (Second)	Storage Life (Days)	Samples	References
1	AChE-ChO/GO-IL/GCE	ADPSV	Adsorption	8.85 × 10^−4^, 1.352 × 10^−3^	5 × 10^−3^–1 × 10^−6^	7.4	NR	NR	90	Serum	[7]
2	AChE-ChO/GO-AuNPs-CS/Fe_3_O_4_–TiO_2_@NH_2_/GCE	ECL	Cross-linking	0.002.2	6.7 × 10^−3^ –0.92 × 10^3^	8	NR	NR	15	Serum	[8]
3	AChE-ChO/QDs/rGO/GCE	ECL, CV	Cross-linking	8.8, 4.7	10–210, 10–250	9.0	NR	NR	7	Serum	[9]
4	AChE-ChO/a PDDA/ZnO/MWCNTs/PGE	CV	Adsorption	0.3	1.0–0.8 × 10^3^, 1.0–10^3^	NR	50	60	90	Serum	[10]
5	AChE-ChO/c PPy-PVS/Pt	CV	Cross-linking	5.0 × 10^−3^	10^−5^–10^−3^	9.0	65	200	NR	Artificial blood	[11]
6	AChE-ChO/MWCNT-MnO_2_/rGO/Au	CV	Cross-linking	0.1	0.1–1.00	7.4	35	NR	90	Serum	[12]
7	AChE-ChO/MOF/PtNPs/Au	DPV	Covalent attachment	0.01	0.01–500	7.4	30	NR	120	Serum	[13]
8	AChE-ChO/CS/Fe@AuNPs/Au	SWV	Cross-linking	5 × 10^−2^	5.0 × 10^−3^–400	7.0	30	3	90	Serum	[14]
9	AChE-ChO/rGO/PtNP/ITO	CV	Cross-linking	5 × 10^−3^	5.0 × 10^−3^−700	7.0	35	4	120	Serum	[15]
11	AChE-ChO/Fe_2_O_3_/rGO/PEDOT/FTO	EIS	Cross-linking	4.0 × 10^−3^	0.004–800	7.0	30	3	90	Serum	[16]
12	AChE-ChO/AuNPs/pTTB/SPCE	CA	Covalent attachment	0.0026	0.7 × 10^−3^ −1.5 × 10^3^	NR	NR	NR	NR	Serum	[17]
13	AChE-ChO/CDs-APTES/CPE	CV	Cross-linking	5.0 × 10^−3^	10^−5^−10^−2^	7.0	60	200	NR	Artificial blood	[18]
14	AChE/PANI-Nano-ZSM−5/GCE	SWV	Entrapment	0.1	1.0–10^3^	7.4	NR	NR	15	-	[33]
15	AChENPs/ChONPs/GONS/PtNPs/PGE	CV	Adsorption	0.001	0.001–200	7.5	35	3	180	Serum	This work

## Data Availability

All authors declare the availability of the data. We leave it up to the publisher.
